# Exosomal lncRNA ZFAS1 regulates esophageal squamous cell carcinoma cell proliferation, invasion, migration and apoptosis via microRNA-124/STAT3 axis

**DOI:** 10.1186/s13046-019-1473-8

**Published:** 2019-11-27

**Authors:** Zhirong Li, Xuebo Qin, Wei Bian, Yishuai Li, Baoen Shan, Zhimeng Yao, Shujun Li

**Affiliations:** 1 Clinical Laboratory Center, The Second Hospital of Hebei Medical University, Shijiazhuang, 050000 Hebei Province People’s Republic of China; 2 Department of Thoracic Surgery, Hebei Provincial Chest Hospital, Shijiazhuang, 050000 Hebei Province People’s Republic of China; 3 Department of Hepatobiliary Surgery, The Second Hospital of Hebei Medical University, Shijiazhuang, 050000 Hebei Province People’s Republic of China; 4 Research Center, The Fourth Hospital of Hebei Medical University, Shijiazhuang, 050000 Hebei Province People’s Republic of China; 5 Institute of Precision Medicine and Pathology, Jinan University, Guangzhou, 510000 Guangdong Province People’s Republic of China; 6 Department of Thoracic Surgery, The Second Hospital of Hebei Medical University, No.215 Heping West Road, Shijiazhuang, 050000 Hebei Province People’s Republic of China

**Keywords:** Esophageal squamous cell carcinoma, Exosomes, LncRNA ZFAS1, microRNA-124, STAT3, Proliferation, Migration, Invasion, Apoptosis

## Abstract

**Background:**

In recent years, long non-coding RNAs (lncRNAs) are of great importance in development of different types of tumors, while the function of lncRNA ZFAS1 is rarely discussed in esophageal squamous cell carcinoma (ESCC). Therefore, we performed this study to explore the expression of exosomal lncRNA ZFAS1 and its molecular mechanism on ESCC progression.

**Methods:**

Expression of ZFAS1 and miR-124 in ESCC tissues was detected. LncRNA ZFAS1 was silenced to detect its function in the biological functions of ESCC cells. A stable donor and recipient culture model was established. Eca109 cells transfected with overexpressed and low expressed ZFAS1 plasmid and miR-124 inhibitor labeled by Cy3 were the donor cells, and then co-cultured with recipient cells to observe the transmission of Cy3-ZFAS1 between donor cells and recipient cells. The changes of cell proliferation, apoptosis, invasion, and migration in recipient cells were detected. The in vivo experiment was conducted for verifying the in vitro results.

**Results:**

LncRNA ZFAS1 was upregulated and miR-124 was down-regulated in ESCC tissues. Silencing of ZFAS1 contributed to suppressed proliferation, migration, invasion and tumor growth in vitro and induced apoptosis of ESCC cells. LncRNA ZFAS1 was considered to be a competing endogenous RNA to regulate miR-124, thereby elevating STAT3 expression. Exosomes shuttled ZFAS1 stimulated proliferation, migration and invasion of ESCC cells and restricted their apoptosis with increased STAT3 and declined miR-124. Furthermore, in vivo experiment suggested that elevated ZFAS1-exo promoted tumor growth in nude mice.

**Conclusion:**

This study highlights that exosomal ZFAS1 promotes the proliferation, migration and invasion of ESCC cells and inhibits their apoptosis by upregulating STAT3 and downregulating miR-124, thereby resulting in the development of tumorigenesis of ESCC.

## Background

Esophageal cancer is the sixth primary cause of cancer mortality and the eighth commonest cancer type in the world [1]. Nearly 330,000 new cases are reported worldwide each year, and more than 270,000 people die of esophageal cancer each year [2] Especially, esophageal cancer is prevalent in China, and the main subtype of esophageal cancer is esophageal squamous cell carcinoma (ESCC) [3]. Drugs are considered to be effective in the treatment of ESCC, including platinums, taxanes, irinotecan, fluoropyrimidines and targeted therapies [4]. The main risks factors of ESCC are alcohol and tobacco use, but other conditions that cause chronic irritation of esophageal mucosa could also increase the risk of ESCC [5]. Diagnosis and treatment of ESCC has a significant improvement, but the 5-year survival rate of patients undergoing treatment is only about 25–30% [6]. Therefore, there is an urgent need to explore an accurate therapeutic target for treatment of ESCC.

Long non-coding RNA (lncRNA) is a class of non-coding RNA which does not encode protein coding potential at 200 bases. Through different mechanisms, lncRNAs play a role in development, differentiation, and tumor formation [7]. LncRNA ZFAS1 is a newly discovered lncRNA which is downregulated in human breast cancer, and it may serve as a cancer-inhibiting factor [8]. A previous study has demonstrated that lncRNA ZFAS1 expression was highly expressed in ESCC tissues, and the high expression of ZFAS1 in ESCC indicates a poor survival of patients [3]. Exosomes mediate the cellular communications in cancer through transmitting active molecules. A study showed that lncRNA ZFAS1 mediated by exosomes promotes the progress of gastric cancer [9]. MicroRNAs (miRNA) could adjust the expression of gene, and are small and non-coding RNA molecules; miR-124 is the richest miRNAs in the brain [10]. Interestingly, a previous research has demonstrated that ESCC risks may be altered by the miR-124 polymorphism in the Chinese Kazakh population [11]. One of the direct targets of miR-124 is signal transducer and activator of transcription 3 (STAT3) [12]. STAT3 is one of the members of the cytosolic transcription factor family, and it is a large transcription factor of 92-kDa, whose gene is placed in chromosome 17q21 [13, 14]. There is a study demonstrated that miR-143 may inhibit the proliferation, invasion and metastasis of ESCC by down-regulating STAT3 [15]. The main objective is to investigate the effect of exosomal ZFAS1 on the growth activity, invasion and migration of ESCC cells and its molecular mechanisms.

## Materials and methods

### Ethics statement

The study was approved by the Ethics Committee of The Second Hospital of Hebei Medical University and informed written consent was obtained from all patients. All animal experiments were in line with the Guide for the Care and Use of Laboratory Animal by International Committees.

### Study subjects

A total of 136 ESCC patients (mean age of 66.18 ± 7.14 years) who received resection in The Second Hospital of Hebei Medical University from June 2015 to June 2018 were collected. There were 92 males and 44 females, and 50 cases with lymph node metastasis (LNM), and 86 cases without LNM. Meanwhile, the corresponding adjacent normal tissues were taken as the control group. Those patients who have undergone radiotherapy and chemotherapy, patients with infection phenomena, patients who have history of other malignant tumors except for ESCC or patients who have primary immunodeficiency and acquired immure deficiency syndrome (AIDS), were excluded from this study.

### Screening of cell lines

Five ESCC cell lines EC9706, Eca109, TE-13, TE-1 and TTN were purchased from the Cell Bank of Type Culture Collection of Chinese Academy of Sciences (Shanghai, China). The five cell lines were cultured in medium and renewed every 24 h for subculture. The cell lines with the highest expression of ZFAS1 were screened out for subsequent experiments by reverse transcription quantitative polymerase chain reaction (RT-qPCR). The cells were seeded in 6-well plates at the density of 3 × 10^5^ cells/well. When the cell confluence reached 80%, lipofectamine 2000 (Invitrogen, Carlsbad, California, USA) kit was used for transfection. Four μg target plasmid and ten μL lipofectamine 2000 was diluted by 250 μL serum-free Opti-MEM (Gibco, Carlsbad, California, USA) medium, respectively. After being placed for 20 min, the mixed solution was added to the culture well, and then cultured with 5% CO_2_. After 6 h, it was replaced to complete culture medium, and the cells were collected after culturing for 48 h.

### Exosomes separation

The ESCC cells post transfection were seeded in RPMI 1640 medium containing 10% fetal bovine serum (FBS) without exosomes and cultured in a cell culture box at 37 °C with 5% CO_2_. After 3 days, cell supernatants were collected and centrifuged to remove cell fragments. According to the Hieff™ Quick exosome isolation kit (article number: 41201ES50, Shanghai YEASEN Biotechnology Co., Ltd., Shanghai, China. The kit was a rapid extraction kit for cell culture supernatant, which was suitable for the extraction of supernatant of the cell culture medium, the urine, or the ascites), the exosomes were extracted. The centrifuged cell supernatant and exosomes separation reagent were centrifuged in the eppendorf (EP) tube at 10,000 g, 4 °C for 1–2 h. The precipitates were the exosomes. According to the ratio of the volume of initial culture medium and the suspension (10: 1), exosomes were resuspended with phosphate buffered saline (PBS). The resuspended exosomes (30 μL) were added with equal volume of radioimmunoprecipitation assay (RIPA) lysate into EP tubes, mixed and placed on ice. The exosomes was continuously lysed by a microwave for 2 times, 10 s each time. And then, the concentration of protein in exosomes was measured by bicinchoninic acid (BCA) quantitative kit (Beyotime Biotechnology, Nantong, China). Exosomes identification: the exosomes marker (CD63, CD9, CD81) was detected by western blot analysis and the morphology of exosomes was observed by a transmission electron microscope (TEM) (JEM-1010, JEOL, Tokyo, Japan). Finally, the exosomes secreted by ESCC cells were obtained.

### TEM observation

The exosomes was precipitated and immediately fixed in 2.5% glutaraldehyde at 4 °C. Then the specimens were dehydrated by gradient alcohol and immersed in epoxy resin. The ultra-thin sections were stained with uranyl acetate and lead citrate and observed under a TEM (JEM-1010, JEOL, Tokyo, Japan).

### Fluorescence-labeled exosomes and uptake of the exosomes

Exo-Red fluorescent staining kit (SBI System Bioscience, San Francisco, CA) was used to label the exosomes. The operation process was carried out according to the instructions of the kit. The exosomes were re-suspended with PBS and mixed with Exo-Glow Red, and then incubated for 10 min. Exo Quick-TC reagent was added to terminate the labeling reaction. After the mixture was centrifuged, the supernatant was discarded and exosomes were resuspended with PBS for further use. Cells (1 × 10^5^) were seeded into a 35-mm dish, after the cell adhered to the wall, the labeled exosomes with 100–150 μL were added, cultured for 24 h, and observed under a confocal microscope.

### Cell grouping and transfection

ESCC cell line Eca109 with a well growth in the logarithmic phase was selected to observe the effect of lncRNA ZFAS1 on the growth, invasion and migration of ESCC cells. The cells were distributed into sh-negative control (NC) group (transfected with sh-ZFAS1 plasmid NC) and sh-ZFAS1 group (transfected with sh-ZFAS1 plasmid). In order to explore the effect of ZFAS1 on the biological functions of ESCC cells in the exosomes, a co-culture model was established to investigate the effect of the exosomal ZFAS1 on the recipient cells. Cells were assigned into the overexpressed (Oe)-NC-exo group (donor Eca109 cells transfected with Oe-ZFAS1 plasmid NC labeled by Cy3 was co-cultured with recipient Eca109 cells), the Oe-ZFAS1-exo group (donor Eca109 cells transfected with Oe-ZFAS1 plasmid labeled by Cy3 was co-cultured with recipient Eca109 cells), the sh-NC-exo group (donor Eca109 cells transfected with sh-ZFAS1 plasmid NC labeled by Cy3 was co-cultured with recipient Eca109 cells), the sh-ZFAS1-exo group (donor Eca109 cells transfected with sh-ZFAS1 plasmid labeled by Cy3 was co-cultured with recipient Eca109 cells) the inhibitor NC-exo group (donor Eca109 cells transfected with miR-124 inhibitor NC labeled by Cy3 was co-cultured with recipient Eca109 cells) and the miR-124 inhibitor-exo group (donor Eca109 cells transfected with miR-124 inhibitor labeled by Cy3 was co-cultured with recipient Eca109 cells). sh-ZFAS1, sh-NC, Cy3-Oe-ZFAS1, Cy3-Oe-NC, Cy3-sh-ZFAS1, Cy3-sh-NC, Cy3-miR-124 inhibitor and Cy3-inhibitor NC were bought from RiboBio Co., Ltd. (Guangdong, China). The transfection of sh-ZFAS1, sh-NC, Cy3-Oe-ZFAS1, Cy3-Oe-NC, Cy3-sh-ZFAS1, Cy3-sh-NC, Cy3-miR-124 inhibitor and Cy3-inhibitor NC into cells were strictly in accordance with the steps of the kit of Lipofectamine™ RNAiMAX (Invitrogen, Carlsbad, CA, USA).

### Establishment of cell co-culture model

After 36 h transfection of Cy3-Oe-ZFAS1, Cy3-Oe-NC, Cy3-sh-ZFAS1, Cy3-sh-NC, Cy3-miR-124 inhibitor and Cy3-inhibitor NC, Eca109 cells (as donor cells) were collected and inoculated with 1 × 10^5^ cells/well into the apical chamber of Transwell culture plate. Complete medium was replenished to 300 μL. Eca109 cells (as recipient cell) were seeded into the basolateral chamber of Transwell 1 day in advance. The density of the plate in the cell was 1 × 10^5^ cells/well, and 3 parallel wells were set up in each group. After co-culture of apical and basolateral chambers for 24 h, FSX100 organisms could only observe the entry of Cy3-ZFAS1 into recipient cells under a navigator. Meanwhile, the recipient cells were collected and the total RNA was extracted. The expression of ZFAS1 and miR-124 in recipient cells was detected by RT-qPCR.

### 5-ethynyl-2′-deoxyuridine (EdU) assay

Cells (4 × 10^3^ per well) were seeded in 96-well plates and cultured to 80% confluence. Cell proliferation was detected by EdU kit (RiboBio, Guangzhou, China). After removing the original medium, it was incubated with 100 μL 50 μm EdU medium (Dilution of EdU solution with cell medium according to 1000: 1) for 2 h. Cells were fixed with 4% paraformaldehyde (50 μL) for 30 min and incubated with 50 μL 2 mg/mL glycine for 5 min. Cells were incubated with 100 μL 0.5% Triton X-100 osmotic agent per well for 10 min, and incubated in the dark for 30 min with 100 μL of 1× Apollo® staining reaction at room temperature, then infiltrated and decolorized with methanol. Finally, the cells were stained with 4′,6-diamidino-2-phenylindole (DAPI) and examined by a laser confocal microscope (Leica, Carl Zeiss, Jena, Germany).

### Colony formation assay

For colony formation assay, the transfected cells were seeded into 6-well plates at the density of 400 cells per well. After 7–14 days, the culture was terminated after the colonies could be seen by the naked eye. Then the medium was absorbed and washed twice with PBS. Thirty minutes were used to fix the cells by methanol and stained it with 0.1% crystal purple staining solution, and colony imaging was counted. At last, the rate of cell colony formation was calculated.

### Flow cytometry

After 48 h of transfection, the cells were detached with 0.25% trypsin without ethylene diamine tetraacetic acid (EDTA) (PYG0107, Boster, Wuhan, Hubei, China) and collected in a flow tube. The supernatant was discarded by centrifugation. According to the instructions of Annexin-V-fluorescein isothiocyanate (FITC) cell apoptosis detection kit (K201–100, BioVision, Palo Alto, USA), the Annexin-V-FITC, propidium iodide (PI), hydroxyethyl piperazine ethanesulfonic acid (HEPES) buffer solution was prepared into Annexin-V-FITC/PI staining solution at 1: 2: 50. Cells (1 × 10^6^) were resuspended in every 100 μL staining solution, then incubated for 15 min, and 1 mL HEPES buffer was added for mixing. The fluorescence of FITC and PI was detected by 515 nm and 620 nm band pass filters at 488 nm wavelength, and the apoptosis was detected.

### Scratch test

The ESCC cells after transfected for 48 h were seeded into 6-well plates with 5 × 10^5^ cells per well. After the cells were completely adhered to the wall, the cells were scratched with a 2-mm cell scraper in the middle of each well and cultured for 24 h. The cells were photographed at 0 h and 24 h after scratching, and the scratch distance was calculated by Image-Pro plus 6.0.

### Tranwell assay

The transwell chamber coated with matrigel was preheated to 37 °C. After detachment and transfection, the cells were divided into the same groups as above. Serum-free medium was used to wash the cells twice and suspended it. The cell density was adjusted to 1 × 10^5^ cells/mL. RPMI 1640 medium (600 μL) containing 20% FBS was added to the transwell basolateral chamber. Cell suspension with 200 μL was added to the apical chamber of transwell and cultured for 48 h. The transwell chamber was taken out, the cells near the lateral membrane of the apical chamber were wiped off, and the cells were fixed with 4% paraformaldehyde solution for 10 min after washing with PBS, followed by crystal violet staining, and then the cell staining was observed under an optical microscope. Five high power visual fields were randomly selected for cell counting, and 3 parallel wells were set up in each group.

### RNA-fluorescence in situ hybridization (FISH) assay

The subcellular localization of lncRNA ZFAS1 in cells was identified by FISH technique. According to the instructions of Ribo™ lncRNA FISH Probe Mix (Red) (RiboBio Co., Ltd., Guangzhou, China), the specific methods were as follows: the cover glass was putted in the 24-well culture plate, and the cells were inoculated according to 6 × 10^4^ cells/well, so that the confluence of the cells was about 80%. The slides were taken out and fixed at room temperature with 1 mL 4% paraformaldehyde after cleaning with PBS. After being treated with protease K, glycine and acetylation reagent, cells were added with 250 μL prehybridization solution and incubated at 42 °C for 1 h. The prehybridization solution was sucked out, after which cells were hybridized with 250 μL lncRNA ZFAS1 (300 ng/mL) hybrid solution containing probe overnight at 42 °C. After phosphate-buffered saline with Tween (PBST) cleaning for 3 times, the nucleus was stained with DAPI solution (ab104139, 1: 100, Abcam, Shanghai, China) diluted by PBST, and then added to the 24-well culture plate and dyed for 5 min. Finally, cells were sealed with anti-fluorescent quenching agent, and a fluorescence microscope (Olympus, Tokyo, Japan) was used to observe and take pictures.

### Dual luciferase reporter gene assay

The sequence of wild type (WT) of miR-124 and STAT3 mRNA 3′-untranslated region (3′-UTR) and sequence of mutant type (MUT) after site-directed mutation of WT target site were synthesized. pmiR-RB-REPORT™ plasmid (RiboBio Co., Ltd., Guangzhou, China) was digested by restriction endonuclease. Then the synthetic target gene fragments WT and MUT were inserted into pmiR-RB-REPORT™ vector (RiboBio Co., Ltd., Guangzhou, China). At the same time, the empty plasmid was transfected as the control group, and the correct luciferase reporter plasmids WT and MUT were used for subsequent transfection. The vectors of MUT and WT were co-transferred to 293 T cells with mimic-NC or miR-124 mimic together with oe-NC or oe-ZFAS1, respectively. The cells were collected and lysed after 48-h transfection, and the supernatant was obtained by centrifugation for 3–5 min. Luciferase detection kit (RG005, Beyotime Biotechnology Co., Ltd., Shanghai, China) was applied for determining the relative lights units (RLU). Using firefly luciferase as an internal parameter, the RLU value determined by renilla luciferase was divided by the RLU value measured by firefly luciferase, and the relative fluorescence value was obtained.

### RNA immunoprecipitation (RIP) assay

The binding of lncRNA ZFAS1 to Ago2 was detected by RIP kit (Millipore, Bedford, MA, USA). The cells were washed with precooled PBS, and the supernatant was discarded. The cells were lysed by equal volume of ribonuclease inhibitor and protease inhibitor phenylmethylsulphonyl fluoride (PMSF) for 30 min. After centrifuging at 14,000 rpm (4 °C, 10 min), the supernatant was taken out. Part of the cell extract was taken out as Input, another incubated with antibody for co-precipitation. After cleaning, the magnetic beads-antibody complex was re-suspended at 900 μL RIP Wash Buffer and incubated at 4 °C with 100 μL cell extract. The sample was placed on the magnetic pedestal to collect the magnetic bead-protein complex. The samples and Input were detached by protease K and then RNA was extracted for subsequent PCR detection. The antibodies used in RIP were rabbit anti-Ago2 (ab186733, 1:50, Abcam, Shanghai, China). Rabbit anti-IgG (ab109489, 1: 100, Abcam, Shanghai, China) was used as the NC.

### RNA pull-down assay

The cells were transfected with 50 nM biotin labeled WT-bio-miR-124 and MUT-bio-miR-124 (GeneCreate Biological Engineering Co., Ltd. Wuhan, China). Forty-eight hours later, the cells were collected and washed with PBS. The cells were incubated for 10 min in a specific lysis buffer (Ambion, Austin, Texas, USA). The lysate was incubated overnight with M-280 streptavidin beads (S3762, Sigma-Aldrich, St Louis, MO, USA) pre-coated with RNase-free bovine serum albumin (BSA) and yeast tRNA (TRNBAK-RO, Sigma-Aldrich, St Louis, MO, USA) at 4 °C. Cells was washed by precooled pyrolysis buffer twice, low salt buffer for 3 times and once with high salt buffer. The bound RNA was purified by Trizol and then lncRNA ZFAS1 enrichment was detected by RT-qPCR.

### RT-qPCR

After collection and treatment of the cells of each group, the total RNA in cells and tissues were extracted by Trizol (TaKaRa, Dalian, China) method. According to the reverse transcription kit (K1621, Fermentas, Maryland, New York, USA) instructions, cDNA was reversely transcribed by RNA. The ZFAS1, miR-124 and STAT3 primer sequences (Table 1) were designed and synthesized by Shanghai Genechem Co., Ltd. (Shanghai, China). The mRNA expression of each gene was detected according to the requirements of fluorescent quantitative PCR kit (TaKaRa, Dalian, China) by RT-qPCR (ABI 7500, ABI, Foster City, CA, USA). U6 was selected as an internal parameter of miR-124, while glyceraldehyde phosphate dehydrogenase (GAPDH) was selected as the internal parameter of ZFAS1 and STAT3. The 2^-ΔΔCt^ method was used to calculate the relative expression of each target gene.

**Table 1:** Primer sequence

Gene	Primer sequence (5′ – 3′)
ZFAS1	F: 5′-GCGGCCTGGACAACTACTA-3′
	R: 5′-AAGATGGCTTTCGCACCT-3′
miR-124	F: 5′-AAGCTCATCGACTTCGGTTC-3′
	R: 5′-GAGGATCTCCTCGTCCTGCT-3′
STAT3	F: 5′-ACCAGCAGTATAGCCGCTTC-3′
	R: 5′-GCCACAATCCGGGCAATCT-3′
U6	F: 5′-CTCGCTTCGGCAGCACA-3′
	R: 5′-AACGCTTCACGAATTTGCGT-3′
GAPDH	F: 5′-TCCCATCACCATCTTCCA-3′
	R: 5′-CATCACGCCACAGTTTTCC-3′

Note: F, forward; R, reverse**;** miR-124, microRNA-124; STAT3, signal transducer and activator of transcription 3; GAPDH, glyceraldehyde-3-phosphate dehydrogenase

### Western blot assay

Western blot assay was used to detect the protein content of STAT3. One hundred μL RIPA lysate (R0020, Solaibao Technology Co., Ltd., Beijing, China) was added to a centrifugal tube. According to bicinchoninic acid kit (AR0146, Boster Biological Technology co. Ltd., Wuhan, Hubei, China), the protein concentration was determined and the concentration of each sample was adjusted to 3 μg/μL. The extracted protein was added into the sample buffer and boiled at 95 °C for 10 min, with 30 μg per well; and 10% polyacrylamide gel electrophoresis was used to isolate the protein. The protein was transferred to polyvinylidene fluoride membrane (P2438, Sigma-Aldrich, St Louis, MO, USA), and sealed with 5% BSA (10 L16, Zhongsheng Likang Technology Co., Ltd., Beijing, China) for 1 h. Subsequently, the rabbit anti-STAT3 (ab119352, 1: 5000), CD63 (ab59479, 1: 1000), CD9 (ab2215, 1: 1000), CD81 (ab79559, 1: 1000) (all from Abcam, Cambridge, USA) were added and incubated at 4 °C overnight, and then corresponding goat anti-rabbit secondary antibody (ab6721, 1: 2000, Abcam, Cambridge, USA) was incubated at room temperature for 1 h. The membrane was developed through chemiluminescence reagent with the internal reference of GADPH (ab181602, 1: 10,000, Abcam, Cambridge, USA) by using Gel Doc EZ imager (Bio-rad, California, USA). Eventually, the gray value of the target band was analyzed by the Image J software.

### Tumor xenografts in nude mice

A total of 20 female BALB/c nude mice aged 4–6 weeks and weighed 16–22 g were purchased from the Animal research center of Chinese Academy of Sciences (Shanghai, China). All nude mice were randomly grouped (*n* = 5): sh-NC group, sh-ZFAS1 group or Oe-NC-exo group and Oe-ZFAS1-exo group. Each group of nude mice was injected with ESCC cells, and the single cell suspension (4 × 10^6^ cells/mL) was injected subcutaneously into the back of nude mice with a disposable aseptic syringe. After injection, all the nude mice were raised in the animal experimental center. The length diameter (L), width diameter (W) and tumor weight of tumor blocks were measured with a vernier caliper and an electronic balance on the 7th day after injection, and the measurement was conducted once every 7 d. The tumor volume was reckoned as V = W2 × L × 0.52 [16]. The nude mice were euthanized 35 d later, the transplantation tumor was taken out, and the maximum surface of the tumor tissue of nude mice in each group was cut off avoiding the necrotic tissue. The overmuch parts were immersed in 4% neutral formaldehyde solution for overnight fixation and embedded in paraffin. Each wax block was separated with a interval of 50 μm, and 10 sections with thickness of 5 μm were cut continuously for subsequent experiment [17].

### Statistical analysis

All data were analyzed by SPSS 21.0 software (IBM-SPSS Statistics, Chicago, IL, USA). The measurement data were expressed as mean ± standard deviation. Comparisons between two groups were conducted by t-test, while comparisons among multiple groups were assessed by one-way analysis of variance (ANOVA) followed by Tukey’s post hoc test, Pearson analysis was utilized to carry out the correlation analysis. *p* value < 0.05 was indicative of statistically significant difference.

## Results

### LncRNA ZFAS1 is up-regulated and miR-124 is down-regulated in ESCC tissues

**Fig. 1:**
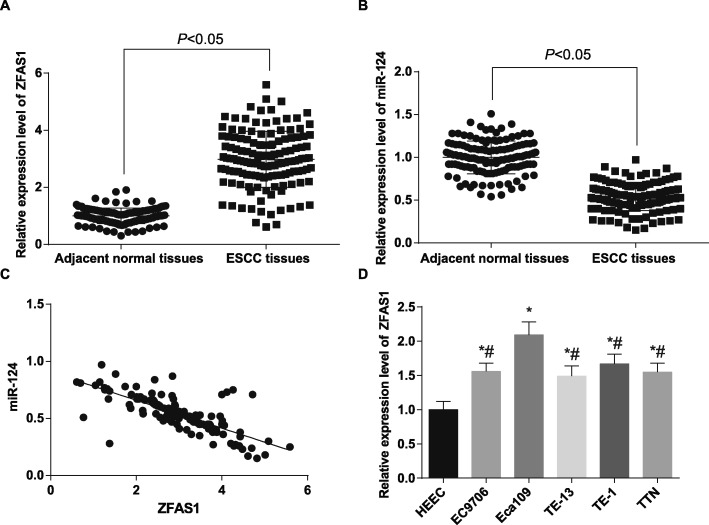
ZFAS1 is highly expressed and miR-124 is lowly expressed in ESCC. **a**: Detection of ZFAS1 expression in ESCC tissues and their adjacent normal tissues by RT-qPCR (*n* = 136). **b**: Detection of miR-124 expression in ESCC tissues and their adjacent normal tissues by RT-qPCR (n = 136). **c**: Correlation between ZFAS1 and miR-124 expression in ESCC tissues analyzed by Pearson correlation analysis. **d**: RT-qPCR detected ZFAS1 expression in human normal esophageal epithelial cells HEEC and five ESCC cell lines. * *p* < 0.05 vs. HEEC cells. # *p* < 0.05 vs. Eca109 cells. Measurement data were depicted as mean ± standard deviation, comparisons between two groups were conducted by independent sample *t*-test, and comparisons among multiple groups were assessed by one-way analysis of variance followed by Tukey’s post hoc test. Repetitions = 3 in cellular experiment

Initially, the expression of ZFAS1 and miR-124 was detected in 136 cases of ESCC tissues and its corresponding adjacent normal tissues. The results of RT-qPCR showed that ZFAS1 expression was higher and miR-124 expression was lower in ESCC tissues than that in adjacent normal tissues (both *p* <  0.05). The results of the correlation analysis of ZFAS1 expression and miR-124 expression in ESCC tissues reported that ZFAS1 expression and miR-124 expression were negatively correlated in ESCC tissues (r = − 0.749, *p* <  0.001) (Fig. 1a-c). We then divided ESCC patients into high expression group (*n* = 69) and low expression group (*n* = 67) on the basis of the median values of ZFAS1 expression in ESCC tissues to further analyze the relationship between the expression of ZFAS1 and the clinicopathological characteristics of the patients with ESCC, and the expression of the ZFAS1 was found to be independent of the patients’ gender and age, and related to the tumor size, the tumor nodes metastasis stage and the presence or absence of LNM, it meant that patients with tumor size more than 3 cm, at TNM III + IV stage and at the presence of LNM had a higher rate of ZFAS1 overexpression (Table 2). Meanwhile, the expression of ZFAS1 in human normal esophageal epithelial cells HEEC and five kinds of ESCC lines EC9706, Eca109, TE13, TE1 and TTN were detected by RT-qPCR. The results suggested that (Fig. 1d) compared with HEEC cells, the expression of ZFAS1 in five kinds of ESCC cells was increased in varying degrees (all *p* < 0.05). The expression of ZFAS1 in Eca109 cell line was dramatically higher than those in EC9706, TE-13, TE-1 and TTN cell lines, so Eca109 cell line was selected for subsequent experiment.

**Table 2:** Relationship between ZFAS1 expression and clinicopathological features in patients with ESCC

Clinicopathological data	Cases (n)	ZFAS1 expression		*P*
		High expression (*n* = 69)	Low expression (*n* = 67)	
Age (years)				0.386
<60	54	30	24	
≥60	82	39	43	
Gender				0.464
Male	92	49	43	
Female	44	20	24	
Tumor size (cm)				0.001
<3	84	33	51	
≥3	52	36	16	
TNM stage				0.009
I + II	82	34	48	
III + IV	54	35	19	
Lymph node metastasis				< 0.001
Yes	50	36	14	
No	86	33	53	

### Silencing ZFAS1 inhibits proliferation, migration and invasion and promotes the apoptosis of ESCC cells

**Fig. 2:**
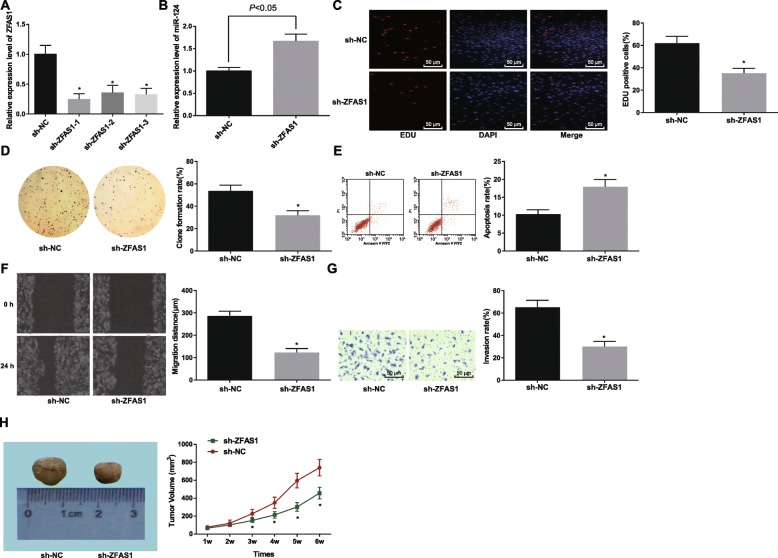
Disturbing the expression of ZFAS1 represses the proliferation, migration and invasion of ESC cells and promote their apoptosis. **a**: Interference efficiency of ZFAS1 in Eca109 cells determined by RT-qPCR. **b**: RT-qPCR detected miR-124 expression in Eca109 cells. **c**: Proliferation of Eca109 cells using EdU assay. **d**: Colony formation ability using colony formation assay. **e**: Apoptosis of Eca109 cells in each group using flow cytometry. **f**: Migration of Eca109 cells in each group using scratch test. **g**: Invasion of Eca109 cells in each group using Transwell assay. **h**: Tumor volume growth curve in each group, *n* = 5. sh-NC group: Eca109 cells transfected with sh-ZFAS1 plasmid NC, sh-ZFAS1 group: Eca109 cells transfected with sh-ZFAS1 plasmid. * *p* < 0.05 vs. the sh-NC group. Measurement data were depicted as mean ± standard deviation; comparisons between two groups were conducted by independent sample *t*-test, and comparisons among multiple groups were assessed by one-way analysis of variance followed by Tukey’s post hoc test; Repetitions = 3 in cellular experiment

In order to observe whether ZFAS1 affects the development of ESCC, we used RT-qPCR to detect the expression of ZFAS1 in Eca109 cells after transfection. The analysis displayed that (Fig. 2a) compared with the sh-NC group, the expression of ZFAS1 in the sh-ZFAS1–1 group, the sh-ZFAS1–2 group and the sh-ZFAS1–3 group were declined, and ZFAS1 expression in the sh-ZFAS1–1 group was the lowest (all *p* < 0.05). Therefore, the sequence in the sh-ZFAS1–1 group was selected to silence ZFAS1 in the following experiments and named it as sh-ZFAS1. RT-qPCR was also utilized to detect miR-124 expression in Eca109 cells after transfected with sh-ZFAS1, and the results demonstrated that (Fig. 2b) in relation to the sh-NC group, miR-124 expression elevated in the sh-ZFAS1 group (*p* < 0.05). The findings of EdU assay, colony formation assay (Fig. 2c-d), scratch test and Transwell assay (Fig. 2f-g) showed decreased proliferation, colony formation, migration, and invasion in cells treated with sh-ZFAS1 (all *p* < 0.05). Besides, flow cytometry results revealed promoted apoptosis in cells treated with sh-ZFAS1 (*p* < 0.05) (Fig. 2e). Tumor xenografts in nude mice tested the effect of sh-ZFAS1 on the growth of tumor in vivo, the results revealed that compared to the sh-NC group, the tumor growth ability in the sh-ZFAS1 group was declined (*p* < 0.05) (Fig. 2h). These findings demonstrated that silencing of ZFAS1 could suppress the proliferation, colony formation, invasion, migration of ESCC cells and tumor growth in vivo but facilitate the cell apoptosis.

### ESCC cells transmit ZFAS1 to surrounding cancer cells through exosomes

**Fig. 3:**
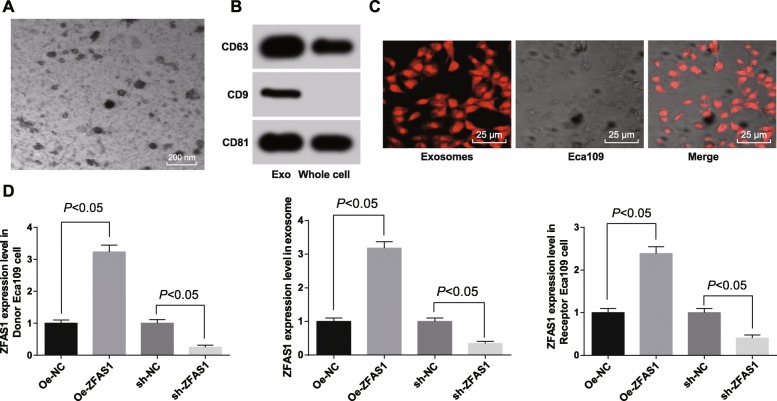
ESCC cells deliver ZFAS1 to surrounding cancer cells by exosomes. **a**: The morphological characteristics of exosomes which was round or oval membranous vesicle were observed by a TEM. **b**: Exosomes surface marker protein CD63, CD9 and CD81 detected by western blot analysis. **c**: Laser confocal observed fluorescence labeled exosomes and exosome uptake (× 400), the expression of ZFAS1 in receptor cells detected by RT-qPCR. **d**: The expression of ZFAS1 in donor Eca109 cells, exosomes and recipient Eca109 cells determined by RT-qPCR. Measurement data were depicted as mean ± standard deviation, and comparisons among multiple groups were assessed by one-way analysis of variance followed by Tukey’s post hoc test; Repetitions = 3 in cellular experiment

We further investigated that the mechanism of the effect of ESCC cells on the surrounding cells. White precipitates were isolated from the culture medium of ESCC cell line Eca109 by differential centrifugation. Under a TEM, the white precipitates were microvesicles with bilayer membrane structure, which were round or oval, and the diameter of vesicles was about 30–60 nm, which was in consistent with the morphological characteristics of exosomes (Fig. 3a). Western blot assay found that the white precipitates had the expression of the exosomes marker (CD63, CD9, CD81) (Fig. 3b). Therefore, we confirmed these white precipitates were the exosomes. The results observed by a laser confocal microscope revealed that exosomes was uptake by Eca109 recipient cells and distributed around the nucleus (Fig. 3c). Therefore, ESCC cells transmit ZFAS1 to surrounding cancer cells through exosomes, which affects the development of ESCC. At the same time, RT-qPCR was utilized to detect the expression of ZFAS1 in donor Eca109 cells, exosomes and recipient Eca109 cells after transfected with Oe-ZFAS1 plasmid and sh-ZFAS1 plasmid. The results showed that (Fig. 3d) ZFAS1 expression elevated in Eca109 donor cells, exosomes and recipient cells relative to that in corresponding NC cells after transfected with Oe-ZFAS1 plasmid (all *p* < 0.05), but declined in Eca109 donor cells, exosomes and recipient cells after transfected with sh-ZFAS1 plasmid (all *p* < 0.05).

### LncRNA ZFAS1 as a competing endogenous RNA (ceRNA) to regulate miR-124

**Fig. 4:**
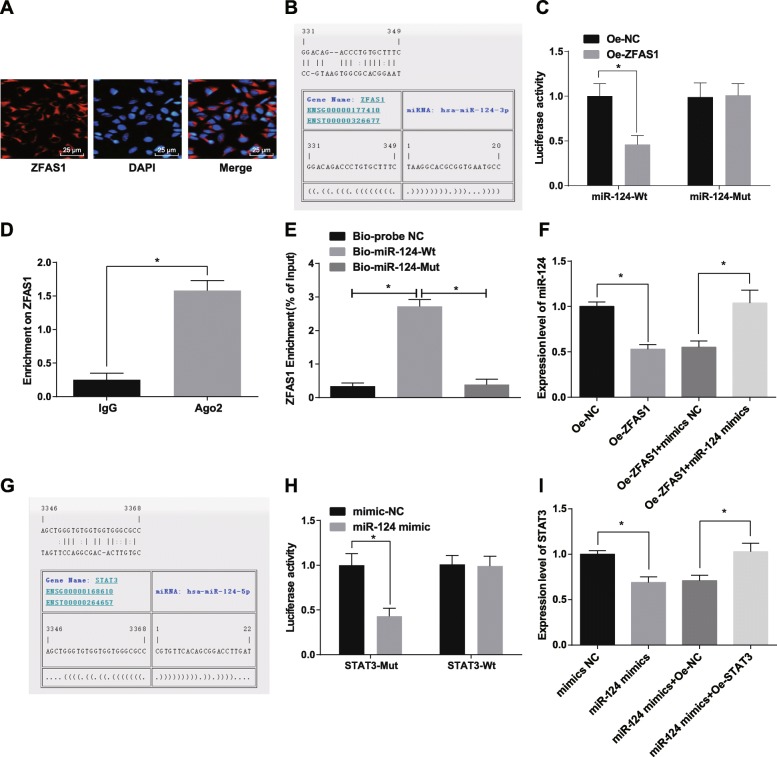
LncRNA ZFAS1 acted as a sponge to regulate miR-124. **a**: FISH assay was performed to verify the ZFAS1 subcellular localization. **b**: Prediction of binding site between ZFAS1 and miR-124 in RNA22 website. **c**: Dual luciferase reporter gene assay was conducted to detect the combination of ZFAS1 and miR-124. **d**: The combination of ZFAS1 and Ago2 detected by RIP assay. **e**: Enrichment of ZFAS1 by miR-124 detected by RNA pull-down assay. **f**: Expression of miR-124 in each group detected by RT-qPCR. **g**: Prediction of binding site between miR-124 and STAT3 in RNA22 website. **h**: The combination of miR-124 and STAT3 detected by dual luciferase reporter gene assay. **i**: STAT3 expression in each group detected by RT-qPCR. * *p* < 0.05. Measurement data were depicted as mean ± standard deviation, comparisons between two groups were conducted by independent sample *t*-test, and comparisons among multiple groups were assessed by one-way analysis of variance followed by Tukey’s post hoc test; Repetitions = 3 in cellular experiment

In order to explore the mechanism of ZFAS1, RNA-FISH was used to detect the subcellular localization of ZFAS1. The results demonstrated that ZFAS1 was concentrated in cytoplasm (Fig. 4a), indicating that ZFAS1 may play a role in cytoplasm. In addition, ZFAS1 could bind to miR-124 (Fig. 4b) through the RNA22 website (https://cm.jefferson.edu/rna22/Precomputed/). The results of dual luciferase reporter gene assay showed that the luciferase activity of WT-miR-124 decreased in cells treated with oe-ZFAS1 (*p* < 0.05). However, there was no significant difference in luciferase activity of MUT-miR-124, which indicating that miR-124 may specifically bind to ZFAS1 (Fig. 4c). The results of RIP assay revealed there was higher specific adsorption level of ZFAS1 on Ago2 (*p* < 0.05) (Fig. 4d). RNA pull-down assay was employed to further verify whether ZFAS1 could be used as the ceRNA to adsorb miR-124, and the results showed that the enrichment level of ZFAS1 in the Bio-miR-124-WT group was significantly higher than that in the Bio-probe NC group (*p* < 0.05). There was no distinct difference in the enrichment level of ZFAS1 in the Bio-miR-124-MUT group and the Bio-probe NC group (*p* > 0.05) (Fig. 4e). The above results suggest that lncRNA ZFAS1 can act as a ceRNA to adsorb miR-124, thereby affecting the expression of miR-124. Additionally, RT-qPCR tested the expression of miR-124 in each group after transfected with Oe-ZFAS1 plasmid and miR-124 mimics. The results presented that in relation to the Oe-NC group, miR-124 expression was degraded in the Oe-ZFAS1 group (*p* < 0.05). In contrast to the Oe-ZFAS1 + mimics NC group, miR-124 expression was raised in the Oe-ZFAS1 + miR-124 mimics group (*p* < 0.05) (Fig. 4f). At the same time, we predicted the target gene of miR-124 on the RNA22 website. It was found that there were binding site between miR-124 and STAT3 (Fig. 4g). Meanwhile, we found that miR-124 bound to STAT3, and STAT3 was the target gene of miR-124 by double luciferase reporter gene assay (Fig. 4h). Meanwhile, RT-qPCR tested the expression of STAT3 in each group after transfected with miR-124 mimics and Oe-STAT3 plasmid. The results presented that in contrast with the mimics NC group, STAT3 expression was degraded in the miR-124 mimics group (*p* < 0.05). In contrast to the miR-124 mimics + Oe-NC group, STAT3 expression was raised in the miR-124 mimics + Oe-STAT3 group (*p* < 0.05) (Fig. 4i). It was speculated that ZFAS1 could inhibit the expression of miR-124, thus up-regulating the expression of STAT3.

### Exosomes shuttle ZFAS1 to promote the proliferation, migration and invasion of ESCC cells, and inhibiting the apoptosis by down-regulating the miR-124 and upregulating the expression of STAT3

**Fig. 5:**
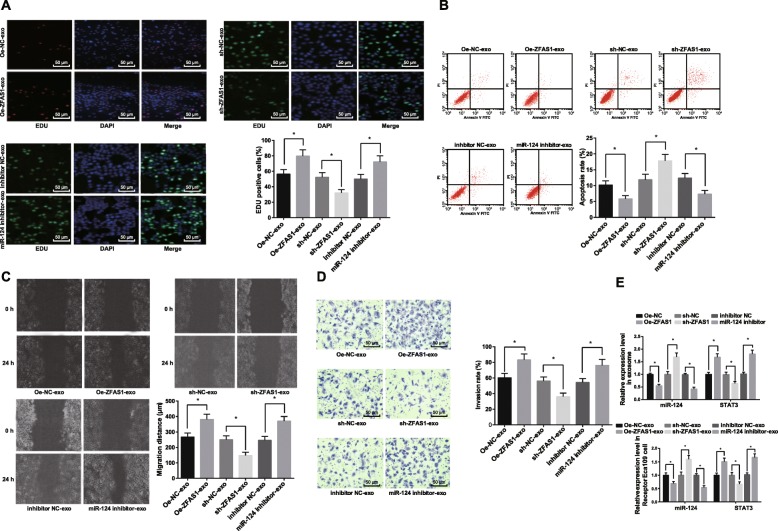
Exosomes shuttle ZFAS1 down-regulates miR-124 and upregulates STAT3 to promote the proliferation, migration and invasion of ESCC cells, and inhibiting the apoptosis. **a**: Proliferation of cells using EdU assay. **b**: Detection of apoptosis in each group by flow cytometry. **c**: Migration of cells using scratch test. **d**: Invasion of cells using Transwell assay. **e**: Expression of miR-124 and STAT3 in exosomes and receptor cells after overexpression of ZFAS1-exo or low expression of miR-124-exo. The Oe-NC-exo group: donor Eca109 cells transfected with Oe-ZFAS1 plasmid NC labeled by Cy3 was co-cultured with recipient Eca109 cells; the Oe-ZFAS1-exo group: donor Eca109 cells transfected with Oe-ZFAS1 plasmid labeled by Cy3 was co-cultured with recipient Eca109 cells; the sh-NC-exo group: donor Eca109 cells transfected with sh-ZFAS1 plasmid NC labeled by Cy3 was co-cultured with recipient Eca109 cells; the sh-ZFAS1-exo group: donor Eca109 cells transfected with sh-ZFAS1 plasmid labeled by Cy3 was co-cultured with recipient Eca109 cells; the inhibitor NC-exo group: donor Eca109 cells transfected with miR-124 inhibitor NC labeled by Cy3 was co-cultured with recipient Eca109 cells; the miR-124 inhibitor-exo group: donor Eca109 cells transfected with miR-124 inhibitor labeled by Cy3 was co-cultured with recipient Eca109 cells. * *p* < 0.05. Measurement data were depicted as mean ± standard deviation, comparisons between two groups were conducted by independent sample t-test, and comparisons among multiple groups were assessed by one-way analysis of variance followed by Tukey’s post hoc test; Repetitions = 3 in cellular experiment

As shown in Fig. 5a-e, the circumstance and effect of exosomal ZFAS1 shuttling into recipient cells was studied by establishing a co-culture model. After co-culture of donor cells and recipient cells with Cy3-labeled Oe-ZFAS1 plasmid, sh-ZFAS1 plasmid or miR-124 inhibitor for 24 h, the effects of exosomal ZFAS1 on proliferation, apoptosis, migration and invasion of ESCC cells were detected. It presented that proliferation, migration and invasion of ESCC cells in the Oe-ZFAS1-exo group increased, while the apoptosis of cells decreased relative to the Oe-NC-exo group (all *p* < 0.05). In relation to the sh-NC-exo group, proliferation, migration and invasion of cells declined and apoptosis raised in the sh-ZFAS1-exo group (all *p* < 0.05). RT-qPCR was adopted to measure the expression of miR-124 and STAT3 in exosomes and recipient cells after treatment of Oe-ZFAS1-exo and sh-ZFAS1-exo, which demonstrated that miR-124 down-regulated and STAT3 up-regulated in exosomes and recipient cells after donor cells with overexpressed ZFAS1 and co-cultured with recipient cells, the trend was apposite in the sh-ZFAS1-exo group and the Oe-ZFAS1-exo group. By comparison with the inhibitor NC-exo group, cell proliferation, migration and invasion ability increased while apoptosis decreased in the miR-124 inhibitor-exo group (all *p* < 0.05). RT-qPCR detected the expression of miR-124 and STAT3 after miR-124 inhibition in exosomes and recipient cells. The results suggested that the donor cells with miR-124 inhibition were co-cultured with the recipient cells, and miR-124 down-regulated and STAT3 up-regulated in exosomes and recipient cells. The obtained data proved that exosomal ZFAS1 may up-regulate STAT3 and down-regulate miR-124 to promote the proliferation, migration and invasion of ESCC cells, inhibit their apoptosis, and then lead to occurrence and development of ESCC tumor.

### Elevated ZFAS1-exo promotes tumor growth in nude mice

**Fig. 6:**
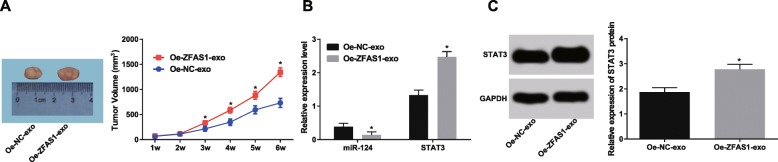
The growth of nude mice in vivo can be facilitated by overexpression of ZFAS1-exo. **a**: Tumor volume growth curve in each group. **b**: Detection of miR-124 and STAT3 expression by RT-qPCR. **c**: Western blot analysis was adopted to verify STAT3 protein expression. *n* = 5. * *p* < 0.05 vs. the Oe-NC-exo group. Measurement data were depicted as mean ± standard deviation, comparisons between two groups were conducted by independent sample t-test

As shown in Fig. 6a, compared with the Oe-NC-exo group, the tumor growth ability in the Oe-ZFAS1-exo group increased significantly from the 3rd week (*p <* 0.05). RT-qPCR revealed that the expression of miR-124 in the Oe-ZFAS1-exo group was notably reduced, while the expression of STAT3 was up-regulated (both *p <* 0.05) relative to the Oe-NC-exo group (Fig. 6b). The results of western blot assay showed that the expression of STAT3 in the Oe-ZFAS1-exo group was significantly higher than that in the Oe-NC-exo group (*p <* 0.05) (Fig. 6c). Taken together, overexpression of ZFAS1-exo can promote the growth of nude mice in vivo.

## Discussion

ESCC is caused by a variety of genetic factors which include miRNA dysregulation and single nucleotide polymorphisms (SNPs), and environmental risks as smoking, folate deficiency, hot tea drinking, and alcohol consumption [11]. There is a study reported that lncRNA ZFAS1 expression was elevated in ESCC tissues [3]. Also, a study provides new insights about the correlation between ESCC risks that in the Chinese Kazakh population and miR-124 polymorphisms, and the ESCC risks might be altered by miR-124 polymorphism [11]. Xuan et al. have suggested that STAT3 may up-regulate MMP2 in ESCC to promote tumor metastasis [18]. Although the combination of surgery, radiotherapy and chemotherapy has improved, the results of ESCC have been disappointing, especially the metastatic ESCC [19]. As the related mechanisms of ZFAS1 in ESCC remains to be excavated, the aim of the present study was to explore the effect of exosomal ZFAS1 on the progression of ESCC and its molecular mechanisms, so as to provides a new idea for further studying the pathogenesis of ESCC. Collectively, our study suggested that exosomal ZFAS1 may up-regulate STAT3 to promote the proliferation, migration and invasion of ESCC cells and inhibit their apoptosis through down-regulating miR-124, thereby causing the development of tumorigenesis of ESCC.

Based on our findings, lncRNA ZFAS1 was up-regulated and miR-124 expression was down-regulated in ESCC. Consistent with our study, lncRNA ZFAS1 was associated with poor prognosis and up-regulated in glioma [8]. In a study, it showed that ZFAS1 was frequently elevated in hepatocellular carcinoma [20], which is in line with our results. Additionally, the finding from our investigation showed that silencing of ZFAS1 inhibited proliferation, migration and invasion and promoted the apoptosis of ESCC cells. A study showed that the inhibition of lncRNA ZFAS1 suppressed glioma cells proliferation, migration and invasion in vitro [8]. Another study has verified that silencing of ZFAS1 suppressed the proliferation and the process of cell cycle, invasion, migration and epithelial-mesenchymal transition [21]. A study has revealed a low expression of miR-124-3p in ESCC tissues [22]. Another study has also indicated that miR-124 expression is reduced in ESCC tissues and related to the clinicopathological parameters, suggesting the poor prognosis [23].

In addition, we have found that ESCC cells transmitted ZFAS1 to surrounding cancer cells through exosomes and elevated ZFAS1-exo promoted tumor growth in nude mice. Exosomes are stored in multivesicular bodies (MVBs) and arise from endosome. By fusing with cell membranes, it is released into the environment [24]. It is enclosed within bimolecular lamellar lipid membrane that contains nucleic acids and proteins. Secreted by all cells, they circulate in the blood [25]. Exosomes are selectively absorbed by the surrounding or distal cells and can be reprogrammed to the recipient cells based on their active cargo content [26]. A previous study has demonstrated that through the transfer of ZFAS1, exosomes promoted proliferation and migration of gastric cancer cells [9]. Except that, our study suggested that ZFAS1 could promote the expression of STAT3 by inhibiting miR-124 expression. The data in a study has demonstrated that ZFAS1 is physically bound to miR-150 and may act as a ceRNA [7]. Furthermore, lncRNA MALAT1 was highly expressed in non-small cell lung cancer cells while miR-124 was poorly expressed, and miR-124 was a direct target of MALAT1 [12]. It has been suggested that high level of ZEB1-AS1 may be positively connected with STAT3 activation [27]. Interestingly, a previous research has demonstrated that STAT3 can invoke lncRNA PVT1 transcription and form a feed-back regulatory loop and lncRNA PVT1 activates the STAT3 signaling pathway by interacting with STAT3 protein [28]. These evidences support that exosomes shuttle ZFAS1 to promote the proliferation, migration and invasion of ESCC cells, and inhibiting the apoptosis by down-regulating miR-124 and up-regulating STAT3.

## Conclusion

In conclusion, our findings showed that exosomal ZFAS1 may upregulate STAT3 through downregulating miR-124 to promote the proliferation, migration and invasion of ESCC cells and inhibit their apoptosis, thereby causing the development of tumorigenesis of ESCC. This paper provides a new idea for further investigating the pathogenesis of ESCC. However, we have not analyzed the survival information of ESCC patients, which would be verified in future research.

## Data Availability

Not applicable

## Abbreviations

3′-UTR: 3′-untranslated region
AIDS: acquired immure deficiency syndrome
ANOVA: one-way analysis of variance
BCA: bicinchoninic acid
BSA: bovine serum albumin
DAPI: 4′,6-diamidino-2-phenylindole 2hci
EDTA: ethylene diamine tetraacetic acid
EdU: 5-ethynyl-2′-deoxyuridine
EP: Eppendorf
ESCC: esophageal squamous cell carcinoma
FBS: fetal bovine serum
FISH: RNA-fluorescence in situ hybridization
L: length diameter
lncRNAs: Long non-coding RNAs
LNM: lymph node metastasis
miRNA: MicroRNAs
MUT: mutant type
MVBs: multivesicular bodies
NC: mimic-negative control
PBS: phosphate buffer saline
PBST: phosphate-buffered saline with Tween
PMSF: phenylmethylsulphonyl fluoride
PVDF: Polyvinylidene fluoride
RIP: RNA immunoprecipitation
RIPA: radioimmunoprecipitation assay
RT-qPCR: reverse transcription quantitative polymerase chain reaction
SNPs: single nucleotide polymorphisms
STAT3: signal transducer and activator of transcription 3
W: width diameter
WT: wild type

## References

[1] Salehi M (2013). Meat, fish, and esophageal cancer risk: a systematic review and dose-response meta-analysis. Nutr Rev.

[2] Bray F (2018). Global cancer statistics 2018: GLOBOCAN estimates of incidence and mortality worldwide for 36 cancers in 185 countries. CA Cancer J Clin.

[3] Shi H (2017). Development and validation of nomogram based on lncRNA ZFAS1 for predicting survival in lymph node-negative esophageal squamous cell carcinoma patients. Oncotarget.

[4] Shah MA (2015). Update on metastatic gastric and esophageal cancers. J Clin Oncol.

[5] Rios-Galvez S, Meixueiro-Daza A, Remes-Troche JM. Achalasia: a risk factor that must not be forgotten for esophageal squamous cell carcinoma. BMJ Case Rep. 2015;2015. 10.1136/bcr-2014-204418 PMC4289772 25564630

[6] Bai Y (2017). Plasma microRNA-19a as a potential biomarker for esophageal squamous cell carcinoma diagnosis and prognosis. Biomark Med.

[7] Li T (2015). Amplification of long noncoding RNA ZFAS1 promotes metastasis in hepatocellular carcinoma. Cancer Res.

[8] Gao K (2017). Long non-coding RNA ZFAS1 is an unfavourable prognostic factor and promotes glioma cell progression by activation of the notch signaling pathway. Biomed Pharmacother.

[9] Pan L (2017). Exosomes-mediated transfer of long noncoding RNA ZFAS1 promotes gastric cancer progression. J Cancer Res Clin Oncol.

[10] Zhu F (2014). MicroRNA-124 (miR-124) regulates Ku70 expression and is correlated with neuronal death induced by ischemia/reperfusion. J Mol Neurosci.

[11] Wu F (2018). A genetic variant in miR-124 decreased the susceptibility to esophageal squamous cell carcinoma in a Chinese Kazakh population. Genet Test Mol Biomarkers.

[12] Li S (2018). The lncRNA MALAT1 contributes to non-small cell lung cancer development via modulating miR-124/STAT3 axis. J Cell Physiol.

[13] Zhang Q (2015). STAT3 inhibitor stattic enhances radiosensitivity in esophageal squamous cell carcinoma. Tumour Biol.

[14] Timme S (2014). STAT3 expression, activity and functional consequences of STAT3 inhibition in esophageal squamous cell carcinomas and Barrett's adenocarcinomas. Oncogene.

[15] Liu J (2016). RETRACTED: MiR-143 inhibits tumor cell proliferation and invasion by targeting STAT3 in esophageal squamous cell carcinoma. Cancer Lett.

[16] Kim CJ (2018). Anti-oncogenic activities of cyclin D1b siRNA on human bladder cancer cells via induction of apoptosis and suppression of cancer cell stemness and invasiveness. Int J Oncol.

[17] Rutka JT (1994). Effects of antisense glial fibrillary acidic protein complementary DNA on the growth, invasion, and adhesion of human astrocytoma cells. Cancer Res.

[18] Xuan X (2015). Stat3 promotes invasion of esophageal squamous cell carcinoma through up-regulation of MMP2. Mol Biol Rep.

[19] Lu JC (2008). Extent of prophylactic postoperative radiotherapy after radical surgery of thoracic esophageal squamous cell carcinoma. Dis Esophagus.

[20] Liu F (2017). Long non-coding RNA ZFAS1 correlates with clinical progression and prognosis in cancer patients. Oncotarget.

[21] Xu W (2018). Silencing of lncRNA ZFAS1 inhibits malignancies by blocking Wnt/beta-catenin signaling in gastric cancer cells. Biosci Biotechnol Biochem.

[22] Zeng B (2019). The role of DNMT1/hsa-miR-124-3p/BCAT1 pathway in regulating growth and invasion of esophageal squamous cell carcinoma. BMC Cancer.

[23] Tian Z (2019). Hypermethylation-mediated inactivation of miR-124 predicts poor prognosis and promotes tumor growth at least partially through targeting EZH2/H3K27me3 in ESCC. Clin Exp Metastasis.

[24] Kourembanas S (2015). Exosomes: vehicles of intercellular signaling, biomarkers, and vectors of cell therapy. Annu Rev Physiol.

[25] Melo SA (2015). Glypican-1 identifies cancer exosomes and detects early pancreatic cancer. Nature.

[26] Kalani A, Tyagi A, Tyagi N (2014). Exosomes: mediators of neurodegeneration, neuroprotection and therapeutics. Mol Neurobiol.

[27] Wang Q (2017). LncRNA ZEB1-AS1 contributes to STAT3 activation by associating with IL-11 in B-lymphoblastic leukemia. Biotechnol Lett.

[28] Zhao J (2018). LncRNA PVT1 promotes angiogenesis via activating the STAT3/VEGFA axis in gastric cancer. Oncogene.

